# Effects of Magnetic Fields of up to 9.4 T on Resolution and Contrast of PET Images as Measured with an MR-BrainPET

**DOI:** 10.1371/journal.pone.0095250

**Published:** 2014-04-22

**Authors:** N. Jon Shah, Hans Herzog, Christoph Weirich, Lutz Tellmann, Joachim Kaffanke, Liliana Caldeira, Elena Rota Kops, Syed M. Qaim, Heinz H. Coenen, Hidehiro Iida

**Affiliations:** 1 Institute of Neuroscience and Medicine, INM-4: Medical Imaging Physics, Forschungszentrum Jülich GmbH, Jülich, Germany; 2 Department of Neurology, Faculty of Medicine, JARA, RWTH Aachen University, Aachen, Germany; 3 Institute of Neuroscience and Medicine, INM-5: Nuclear Chemistry, Forschungszentrum Jülich GmbH, Jülich, Germany; 4 Department of Investigative Radiology, National Cardiovascular Center Research Institute, Osaka, Japan; University of Manchester, United Kingdom

## Abstract

Simultaneous, hybrid MR-PET is expected to improve PET image resolution in the plane perpendicular to the static magnetic field of the scanner. Previous papers have reported this either by simulation or experiment with simple sources and detector arrangements. Here, we extend those studies using a realistic brain phantom in a recently installed MR-PET system comprising a 9.4 T MRI-scanner and an APD-based BrainPET insert in the magnet bore. Point and line sources and a 3D brain phantom were filled with ^18^F (low-energy positron emitter), ^68^Ga (medium energy positron emitter) or ^120^I, a non-standard positron emitter (high positron energies of up to 4.6 MeV). Using the BrainPET insert, emission scans of the phantoms were recorded at different positions inside and outside the magnet bore such that the magnetic field was 0 T, 3 T, 7 T or 9.4 T. Brain phantom images, with the ‘grey matter’ compartment filled with ^18^F, showed no obvious resolution improvement with increasing field. This is confirmed by practically unchanged transaxial FWHM and ‘grey/white matter’ ratio values between at 0T and 9.4T. Field-dependent improvements in the resolution and contrast of transaxial PET images were clearly evident when the brain phantom was filled with ^68^Ga or^ 120^I. The grey/white matter ratio increased by 7.3% and 16.3%, respectively. The greater reduction of the FWTM compared to FWHM in ^68^Ga or^ 120^I line-spread images was in agreement with the improved contrast of ^68^Ga or^ 120^I images. Notwithstanding elongations seen in the z-direction of ^68^Ga or ^120^I point source images acquired in foam, brain phantom images show no comparable extension. Our experimental study confirms that integrated MR-PET delivers improved PET image resolution and contrast for medium- and high-energy positron emitters even though the positron range is reduced only in directions perpendicular to the magnetic field.

## Introduction

The development of hybrid MR-PET scanners has recently gained momentum following the announcement of prototype devices by the major manufacturers of clinical imaging equipment. Two major routes have been pursued thus far: the so-called “true hybrids” and the “tandem” machines [1], [2]. As implied by the name, the tandem machines are two separate scanners, with one placed behind the other, and a shared table. The MR and PET measurements are performed consecutively. The development of true hybrids, on the other hand, has taken the much more difficult route of integrating the PET machine inside the bore of the MR magnet, necessitating a major redesign of the PET components [3]–[5]. Although this engineering challenge might be viewed as a disadvantage, it does offer new possibilities in that spatial and temporal coherence is guaranteed. The MR and PET measurements can be performed simultaneously depending on the required study protocol [6]–[8]. In such an instrument, there is another, incidental, advantage; the range of the emitted positron is somewhat more circumscribed because of the presence of a large static magnetic field, as originally proposed by Iida et al [9]. The circumscribed range should yield improved PET image quality in the directions perpendicular to the main magnetic field of the MR magnet. It has also been expected from Monte Carlo simulations that the more important impact of applying a magnetic field is the improvement of image contrast attributed to the confinement of the spreading positrons [9], [10].

The influence of a magnetic field on positron emitters and the anticipated improvement of PET image resolution and image contrast in hybrid, integrated MR-PET scanners due to the confined positron trajectory has been studied in a number of papers since 1986 [9]–[15]. Those studies investigated reduction in positron range using simulations and/or simple experiments. Following the significance gained by PET/CT in oncological imaging, MR-PET is attracting more and more interest as a new bimodal imaging modality driven by the fact that MRI has a better soft tissue contrast than CT and does not use additional ionising radiation. If MRI and PET data can be recorded simultaneously in an integrated, fully-hybrid scanner, MR-PET offers many perspectives for multi-parametric imaging beyond the common combination of functional PET and anatomical MRI [16]–[18]. Moreover, given the different acquisition times for each modality, a number of MRI scans, with different contrasts, sensitivities and anatomical and functional properties, can be acquired during the data acquisition for a PET scan [19]. Importantly, acquisition of the MR and PET datasets is concurrent.

For small animal research, different solutions for combining MRI and PET have been realised [20]. Considering the potentially high PET resolution of these scanners and the concomitant tiny structures in small animals to be investigated, the positron range of tracers such as ^68^Ga, ^15^O, ^124^I, which have higher positron energies than the standard PET-nuclides ^11^C and ^18^F, cannot be neglected as a factor that has a detrimental effect on PET image resolution [21]. Some small-animal MR-PET scanners use high magnetic field strengths (∼7 T) [3], [4] and thus in such scanners a reduction of the positron range can be expected, and consequential improvement of PET image contrast and resolution.

In the last few years, the first true hybrid MR-PET prototype scanners for human brain imaging at 3 T have been developed by Siemens (Siemens AG, Healthcare Sector, Erlangen, Germany) and have been installed in a limited number of centres worldwide [6]–[8], [22]. More recently, a similar type of true hybrid MR-PET for whole-body studies, called the mMR, from the same company became commercially available and has since been installed in a number of centres [1]. Both types of hybrid MR-PET, the 3T MR-BrainPET and the mMR, integrate newly-developed magneto-insensitive PET detectors [5] into Siemens MRI scanners, the 3T MAGNETOM Tim-Trio MR and the 3T Verio MR, respectively. Although the magnetic field of these hybrid scanners is ‘just’ 3 T, it is of enormous interest whether this field leads to an appreciable improvement in the spatial resolution of PET images, especially if medium- or high-energy positron emitters such as ^68^Ga, ^15^O or ^82^Rb are used [13], [23], [24].

In our institute, two identical BrainPET inserts are currently in operation; one in a 3 T MRI scanner and a second in an ultra-high field 9.4 T whole-body MR scanner for humans, currently being constructed jointly with Siemens. Given the above instrumentation, there exists a unique opportunity for us to investigate experimentally the actual improvement in the spatial resolution of PET images as a function of static magnetic field strength. The experimental results, obtained from fully-functional BrainPET scanners operating in magnetic fields, enable comparison with simulation results and/or experimental results obtained from simpler detector units that have been presented previously in the literature.

The results reported herein are from experiments carried out with different phantoms and different positron emitters in a 9.4 T MR-BrainPET. Previous experiments reported by other investigators only made use of point sources [11], [13]. Primarily, they looked at changed positron range in the plane in which the Lorenz force acts on the charged particles, i.e. perpendicular to the direction of the magnetic field. PET images are, however, acquired in three dimensions. In the experimental setup employed here, in addition to point sources and line sources placed in materials of different density, a realistic brain phantom was also used. Under these experimental conditions, it becomes possible to comprehensively explore the issues relevant for determining the extent of improvement of spatial resolution in PET images in future *in vivo* MR-PET applications.

The present study is intended to evaluate quantitatively the effects of an external magnetic field on PET images acquired using a newly-developed PET insert for MRI scanners. In particular, the improvements in spatial resolution and contrast of the PET images were assessed.

## Materials and Methods

The MR component of the 9.4 T MR-BrainPET is a whole-body MR scanner developed in collaboration by Siemens Healthcare (Erlangen, Germany) and the Forschungszentrum Jülich. The magnet has a warm bore of 90 cm and the inner bore of the gradient coil is 60 cm, into which the BrainPET can be inserted. The superconducting 9.4 T magnet is passively shielded with 870 tons of iron. Although detailed previously in several publications [6], [8], [25], for the sake of clarity some of the technical specifications of the BrainPET are described below. The BrainPET consists of 32 cassettes forming a ring with an inner diameter of 36 cm, which matches the outer diameter of the MR head radiofrequency (RF) coils. Each cassette has 6 detector blocks inline whereby each block consists of 12×12 LSO crystals of size 2.5×2.5×20 mm^3^. Light emissions from these crystals are recorded by an array of 3×3 avalanche photodiodes (APDs). The PET volume-of-interest (VOI) covers 19.2 cm in the axial direction. Each detector cassette is RF-shielded separately in such a way that it is transparent for the low frequency switched magnetic fields used for imaging in the MR scanner. Six-millimetre wide gaps exist between the cassettes and 2.5 mm gaps exist between the detector blocks. The feasibility of simultaneous MR-PET measurements with an almost identical BrainPET insert in the 3T field of a Siemens MAGENETOM Trio has been demonstrated in earlier works [6]–[8], [19].

**Figure 1:**
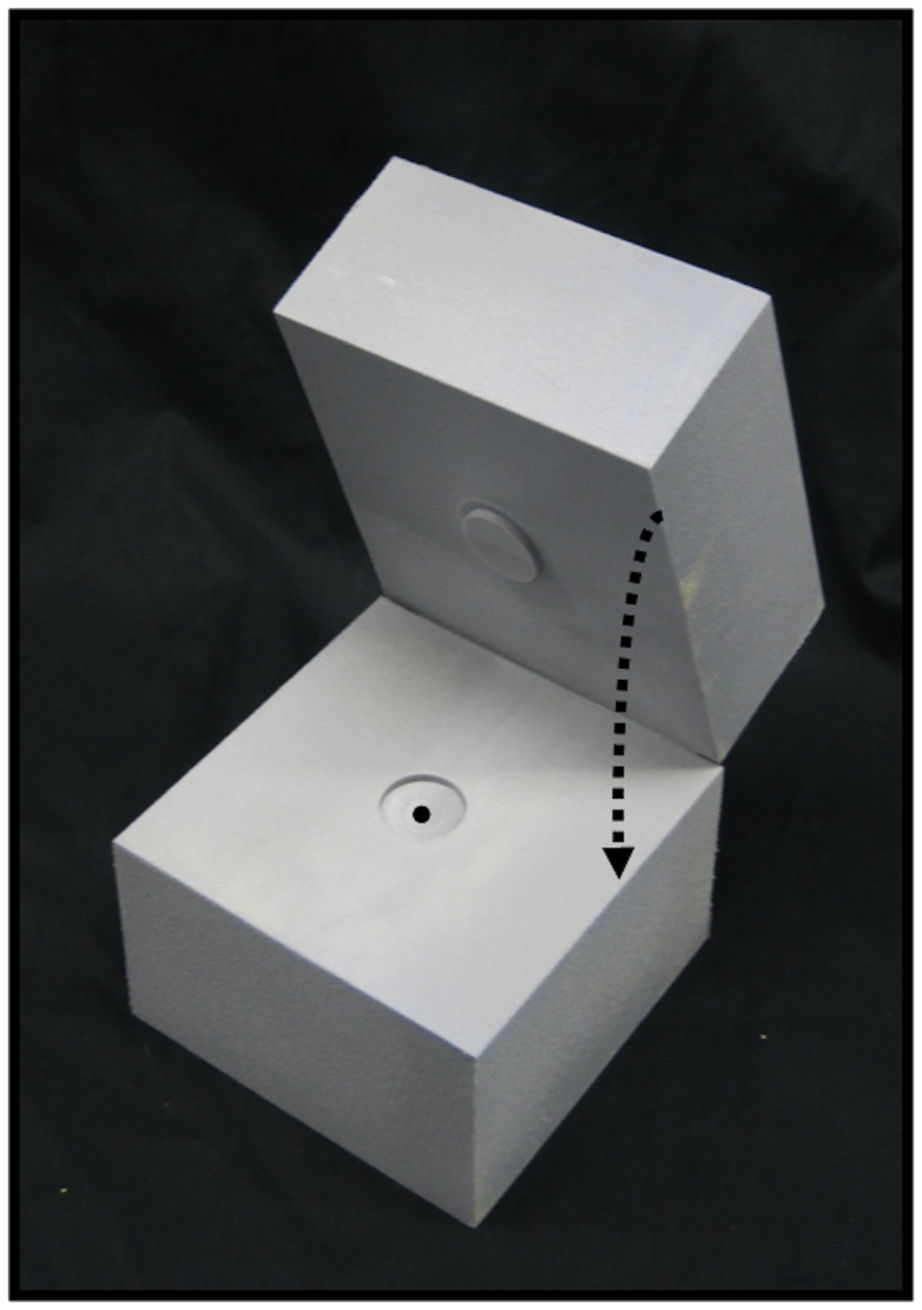
Point source phantom. The block of polyethylene foam consists of two halves whereby on the inner surface of one half, a disk-shaped protrusion fits into a disk-shaped recess on the other. A radioactive drop is placed right in the centre of the cubic block of polyethylene foam with a density of 0.1 g/cm^3^.

Glass capillaries with an inner diameter of 1 mm and an outer diameter of 6 mm were placed in a cylinder of 20 cm length and 20 cm diameter filled with water. The cylinder was positioned within the BrainPET such that the line source was located in and along the central axis of the scanner. Images from a point source of 1 mm^3^ placed in the centre of the BrainPET were measured with the source positioned in the middle of a 10×10×10 cm^3^ block of polyethylene foam with a density of 0.1 g/cm^3^. Given the positrons emitted from the nuclei employed in this study, this density, which is about a third of the density of lung tissue [26], was selected in order to have an expanded positron range, but also to stop the positrons within the realistically-sized phantom. As shown in Fig. 1, the block of polyethylene foam consists of two halves shaped such that a disk-shaped protrusion on the inner surface of one half fits into a disk-shaped recess on the other.

**Figure 2:**
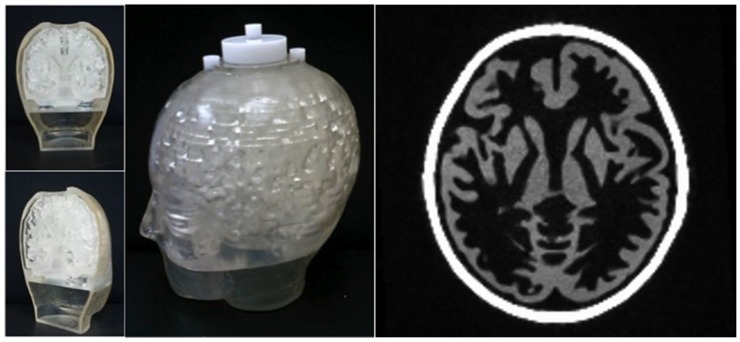
Left: Photographs of the Iida brain phantom used in this study. On the left, the sections show the elaborate internal structure of the phantom with the putative ‘grey/white matter’ compartments. Right: Transaxial MPRAGE image of the brain phantom with the same slice position as shown in Fig. 7.

In order to mimic the human brain, a phantom (subsequently referred to as the “Iida brain phantom”) was employed; the brain phantom (Fig. 2) was constructed from a photo-curable polymer with a density of 1.07 g/mL by using a laser-technique modelling the head shape of a young healthy Japanese volunteer [27]. The Iida brain phantom has two fillable compartments: the outer one, which is to be filled with K_2_HPO_4_ diluted in water (100 g K_2_HPO_4_ and 100 g water), simulates the skull, whereas the inner compartment has the structure of the cortex (grey matter = GM) and can be filled with radioactivity diluted in water. The interior space is solid, i.e. there is no fillable white matter compartment such as in a Hoffman 3D brain phantom [28]. This compartment was used as a reference in the numerical analysis and is denoted in the following as “white matter” (WM). The brain phantom was positioned in the field-of-view (FOV) of the BrainPET such that its centre coincided with the centre of the FOV. The caudal-cranial direction of the BrainPET was parallel to the z-axis of the scanner.

In separate experiments, the phantoms were filled with 3 different positron emitters, the low-energy positron emitter ^18^F, the medium-energy positron emitter ^68^Ga and the high-energy positron emitter ^120^I [29], [30]. ^120^I was chosen because of its high maximum positron energy of and ranges in water of these radionuclides are listed in Table 1. The amount of radioactivity filled into the line source and the point source phantom ranged from 1 to 5 MBq. As indicated above, only the grey matter compartment of the Iida brain phantom was filled with radioactivity; about 30 MBq of each of the different positron emitters was used.

**Table 1:** Literature values of the positron energy in water of ^18^F (14), ^68^Ga (14), and ^120^I (31) and of the positron range (expressed in terms of FWHM and FWTM) in water of ^18^F (14), ^68^Ga (14), and ^120^I (35).

Isotope	Energy		β+ - Range in Water	
	mean	max	FWHM	FWTM
	(keV)	(keV)	(mm)	(mm)
^18^F	248	635	0.1	1
^68^Ga	829	1899	0.5	4
^120^I	1845	4613	4.4	14.6

In Ref. 35 the positron range of ^120^I is reported as the resulting image resolution, if ^120^I is applied in an imaging system with an intrinsic spatial resolution of 1.5 mm FWHM.

**Figure 3:**
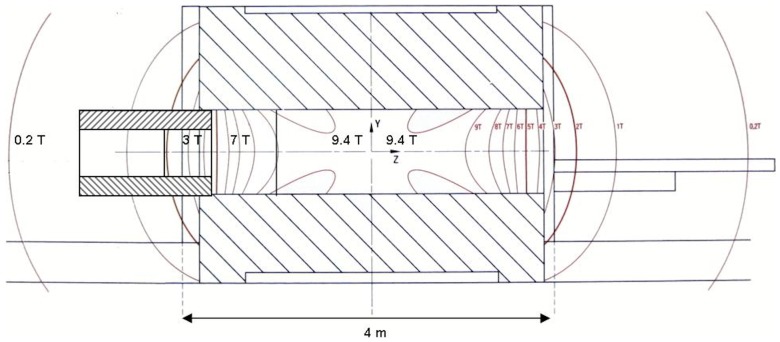
Schematic representation of the magnet and the positions of the PET insert.

In order to examine the influence of different magnetic field strengths, the BrainPET was placed outside the bore of the magnet of the MR scanner and at three positions in the MR scanner. At about 3.5 m behind the scanner, but importantly, outside the 870-ton iron shield the magnetic field is approximately 0 T, that is, 0.022 T to be precise. At the entrance of the bore of the 9.4 T magnet the field is about 3 T (see Fig. 3). Similarly, another point corresponding to 7 T was found and, finally, at the isocentre of the magnet the field has the designed value of 9.4 T. The strength of the magnetic field at the different positions was measured with a Gauss meter. Since no MR acquisitions were performed, there was no need to locate the head RF coil inside the BrainPET insert.

All acquisitions were performed in listmode and framed into sinogram data comprising the total acquisition time prior to reconstruction. In order to take account of radioactive decay, the duration of the individual measurements carried out varied between 600 s and 1000 s.

Point and line spread images were reconstructed using the 3D filtered back-projection algorithm available in the STIR library [32]. Here, a ramp filter (cut-off = 0.5 cycles/pixel), a zoom factor of 2 and an xyz-voxel size of 0.625×0.625×1.25 mm^3^ were selected. Because the point source images showed extensions in x- and z-direction when using ^68^Ga and ^120^I (as detailed below), only the line-spread images were used for the numerical analysis of image resolution in the transaxial (x-y) plane. Vertical and horizontal profiles crossing the lines were extracted from three transaxial images positioned in the middle of the line source and at z-intervals of +/−50 mm. In this way, 6 profiles were evaluated for each radionuclide. The profiles were fitted by Gaussian functions from which the FWHM, as the parameter of image resolution, was obtained. The full width at tenth maximum (FWTM) was obtained directly from the measured profiles using linear interpolation.

Data from the Iida brain phantom were reconstructed using the manufacturer-supplied 3DOSEM algorithm [33] (2 subsets, 32 iterations) taking into account normalization and correction for randoms and attenuation. The resulting image volume had 256×256×153 voxels of 1.25 mm^3^. With ^120^I, as with most other non-standard positron emitters, false coincidences, the so-called gamma-coincidences, occur which cause a flat background so that the conventional scatter correction available in Siemens PET devices does not work properly [34]. Therefore, only the^18^F and ^68^Ga, but not the^120^I images were corrected for scatter.

All reconstructed images were smoothed with a 3D Gaussian filter of 3 mm FWHM. Using the Fusion-tool of PMOD (PMOD Technologies Ltd, Switzerland), the images of the brain phantom filled with ^68^Ga and ^120^I were registered to the ^18^F-images. Regions-of-interest (ROIs) were defined on MPRAGE images of the Iida phantom, which had been registered to the PET images. On five transaxial images, each of 1 cm (8 image planes) separation, ROIs delineating GM and WM were defined by using an isocontour level of 50% of the maximum image intensity. In this way a grey/white (GM/WM) ratio for each image was obtained so that the mean and standard deviation of the GM/WM ratios could be calculated.

Since the magnetic field with its orientation along the z-axis reduces the positron range in the x- and y-direction, but not in the z-direction, it is interesting to look at coronal (x-z-plane) or sagittal (z-y- plane) slices to verify this effect. First, coronal images were examined. Contours around the outer border of the images measured at 0 T and 9.4 T were defined to demonstrate the different changes of image resolution in the x and z direction. In the case of ^18^F and ^68^Ga, the contours were defined at a 20% isocontour level of the image maximum, whereas due to the higher background for ^120^I, a level of 23% had to be applied to obtain contours similar to those of ^18^F and ^68^Ga. Second, in the images of the three radionuclides at 0 T, 3 T, 7 T and 9.4 T, profiles crossing the basal ganglia were defined in the x- and z-directions. Each set of the four profiles was normalized to a common maximum.

## Results

**Figure 4:**
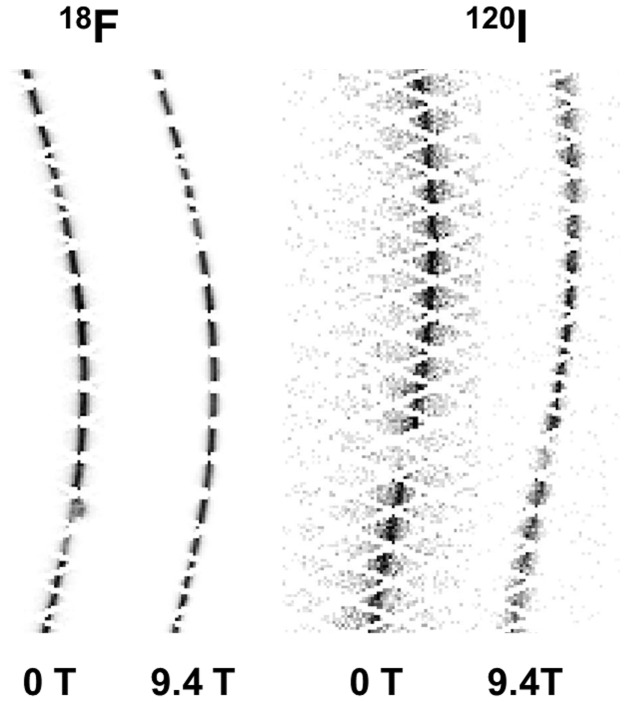
Sinograms of the point source filled with ^18^F or ^120^I and measured at 0 T and 9.4 T. The gap in the sinograms of the ^120^I test were caused by a block failure.

Figure 4 demonstrates the effects of the magnetic field on sinograms of positron emitters with low- and high-positron energy. Whereas no significant difference was observed in the case of ^18^F, there was a clearly discernable effect with a decreased range in the horizontal (radial) direction of the sinogram when the ^120^I-filled point source was immersed in a 9.4 T magnetic field as compared to 0 T.

**Figure 5:**
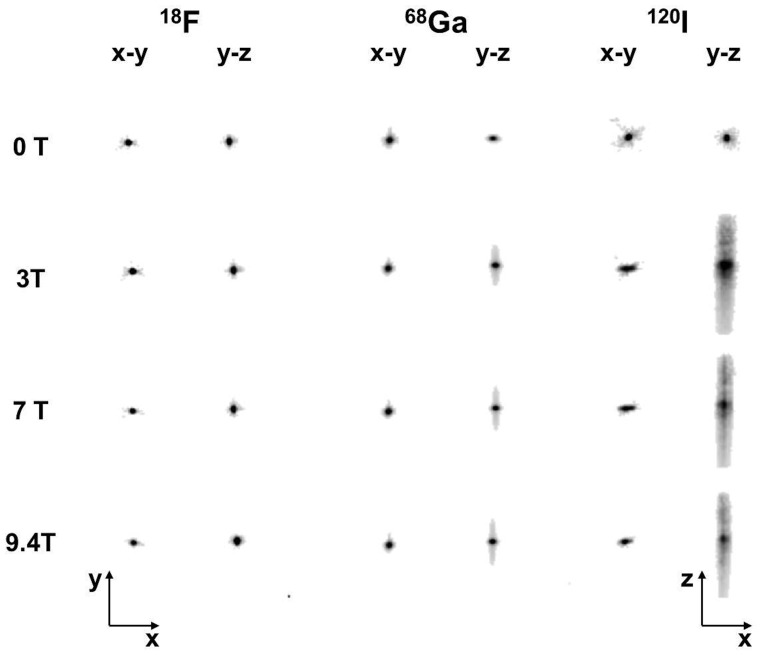
Images of the point source filled with ^18^F, ^68^Ga or ^120^I measured at the four different magnetic field strengths.

Images of the point source located within the polyethylene foam block are shown in Fig. 5. For ^18^F and ^68^Ga, the point images in the x-y plane appear slightly smaller from 0 T to 9.4 T. When the point source contained ^68^Ga or ^120^I, their images showed tails in the x- and z- directions when measured inside the magnetic field. The tails were more pronounced for ^120^I than for ^68^Ga. The tails in z-direction remained unchanged for ≥3 T with a z-FWHM of 4.36±0.04 and 9.86±1.65 for ^68^Ga or ^120^I, respectively. The tails can be explained by the thin gap between the halves of the block at its centre, which is parallel to the x-z-plane (Fig. 1). In this plane medium- and high-energy positrons can spread out in contrast to the vertical (y) direction where they have to interact with the foam. To validate this effect we performed a test in the 3T MR-BrainPET with ^68^Ga and the foam block tilted by 45 degrees around the z-axis. In this case the tails were also tilted by 45 degrees. The spread of the positron distribution is not visible for ^68^Ga or ^120^I, but becomes a clear straight-line distribution along the z axis at >3T, as a result of confinement of positron trajectory in the x-y plane.

**Figure 6:**
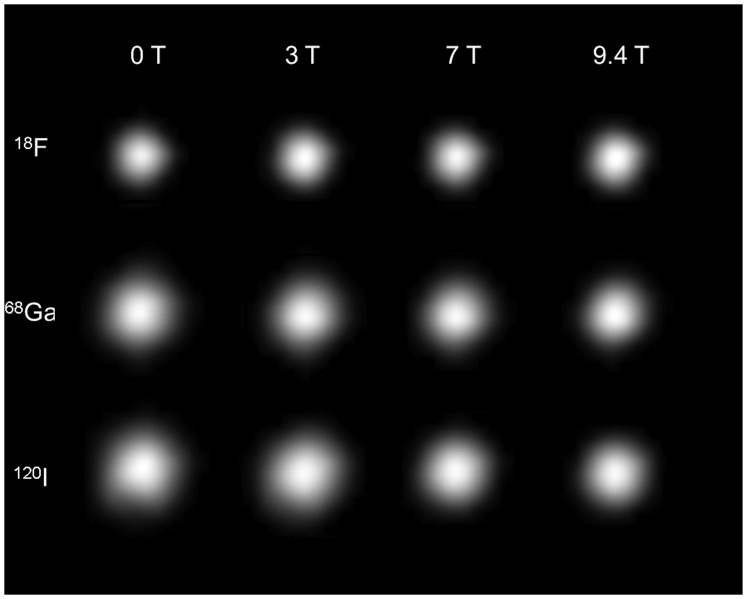
Transaxial PET-images of the glass capillary placed in water, filled with ^18^F, ^68^Ga or ^120^I, and measured at the four different magnetic field strengths.

Figure 6 compares the transaxial images of the line source filled with the three different radionuclides and measured at the four different field strengths. These images demonstrate a decrease of image resolution from low- to high-positron energy. An improvement of the (transaxial) image resolution with increasing magnetic field strength can be observed for ^68^Ga and ^120^I, but not for ^18^F. The numerical results of the line source study are shown in Table 2. Whereas both FWHM and FWTM indicated no effect of the magnetic field for ^18^F, these measures became smaller from 0 T to 9.4 T for ^68^Ga and ^120^I. In agreement with the general cusp-like shape of the distribution of the positron ranges, the numbers of the FWTM show a larger effect with the increasing magnetic field than the FWHM. From 0 T to 3 T the FWHM changed by 0.3%, −3.1% and 1.8% for ^18^F, ^68^Ga and ^120^I, respectively. The corresponding results of the FTWM are −1.6%, −6.1% and −6.7%. Comparing the measurements of ^18^F, ^68^Ga and ^120^I at 0 T and 9.4 T the FWHM was changed by −0.7%, −13.l6% and −14.6%, whereas the FWTM changed by 1.0%, −17.1% and −22.7%.

**Table 2:** Resolution findings expressed as FWHM and FWTM for the three positron emitters studied at 0 T, 3 T, 7 T, and 9.4 T.

	Magnetic Field (T)	FWHM (mm)	FWTM (mm)
**^18^F**	0	2.87±0.09	6.30±0.49
	3	2.87±0.09	6.20±0.47
	7	2.88±0.10	6.29±0.42
	9.4	2.85±0.08	6.36±0.43
**^68^Ga**	0	3.50±0.07	7.46±0.79
	3	3.39±0.07	7.01±0.60
	7	3.22±0.05	6.68±0.61
	9.4	3.02±0.05	6.19±0.53
**^120^I**	0	3.86±0.08	8.62±0.56
	3	3.93±0.13	8.04±0.53
	7	3.60±0.14	7.17±0.46
	9.4	3.30±0.06	6.66±0.41

The data (mean ± standard deviation, n = 6) were calculated from horizontal and vertical profiles crossing the lines sources at three different z-positions.

**Figure 7:**
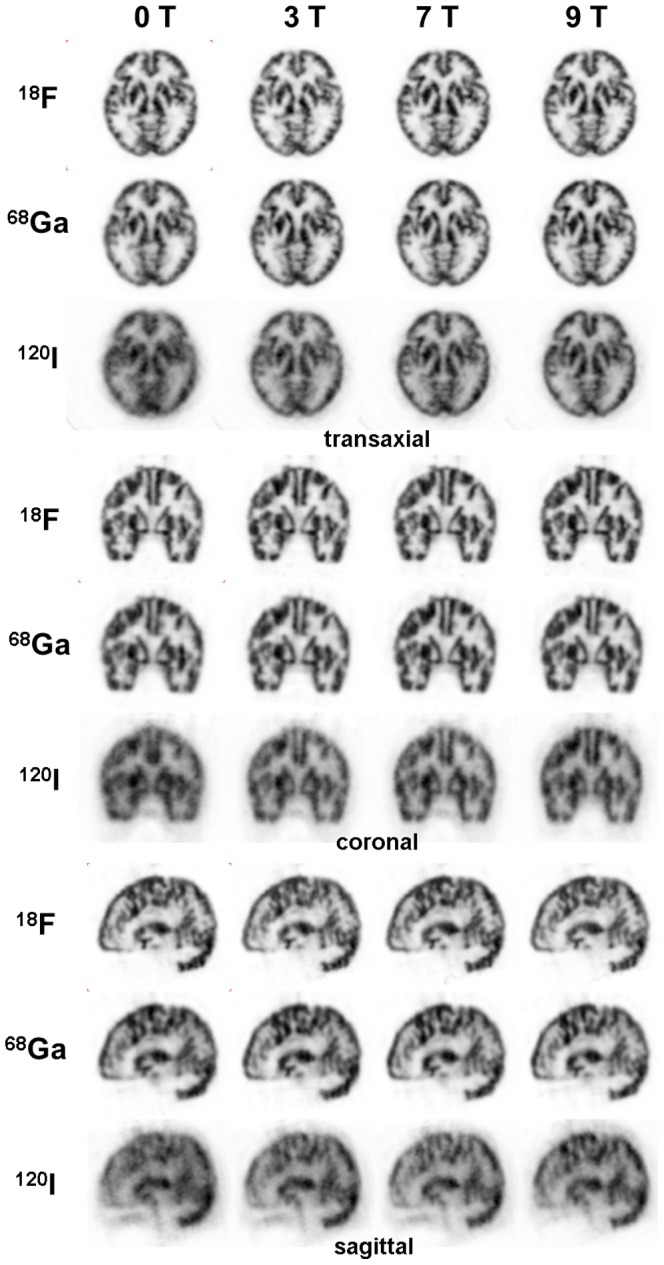
Transaxial, coronal and sagittal PET-images of the Iida brain phantom filled with either of three positron emitters and each measured at 0 T, 3 T, 7 T and 9.4 T. The original reconstructed images are filtered with a 3D Gaussian filter of 3mm FWHM. Each image is scaled to its own maximum. The ^18^F- and ^68^Ga-images are scatter-corrected.

When the Iida brain phantom was filled with ^18^F, there was no visible influence from the magnetic field (Fig. 7). Correspondingly, no improvement of the GM/WM ratio was found when the phantom was scanned in the magnetic field (Table 3).

**Table 3:** Ratios of the reconstructed counts measured in the ‘grey matter’ (GM) and the ‘white matter’ (WM”) compartments of Iida brain phantom filled with ^18^F, ^68^Ga, and ^120^I, respectively.

	Magnetic Field (T)	GM/"WM"
**^18^F**	0	2.71±0.04
	3	2.69±0.05
	7	2.70±0.04
	9.4	2.70±0.05
**^68^Ga**	0	2.32±0.04
	3	2.46±0.05
	7	2.49±0.04
	9.4	2.49±0.05
**^120^I**	0	1.44±0.02
	3	1.59±0.04
	7	1.67±0.04
	9.4	1.68±0.02

The data (mean ± standard deviation) were calculated from GM/WM ratios measured in five transaxial images with z-intervals of 1 cm.

When the brain phantom was filled with ^68^Ga or ^120^I, the influence of the magnetic field became obvious (Fig. 7). A noticeable effect can already be seen at 3 T with an increase of the GM/WM ratio by 6.0% for ^68^Ga and by 10.3% for ^120^I (Table 3). Especially for the high-energy positron emitter ^120^I, the image resolution improves further at 7 T and 9.4 T. For 9.4 T the relative increase of the GM/WM ratio was 7.3% for ^68^Ga and 16.3% for ^120^I.

**Figure 8:**
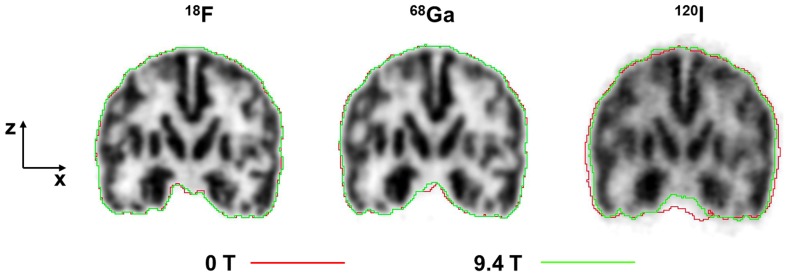
Coronal PET-images of the Iida brain phantom filled with ^18^F, ^68^Ga or ^120^I and measured at 9.4 T. The green contour was defined on these images, whereas the red contour was defined on the corresponding images obtained at 0 T.

**Figure 9:**
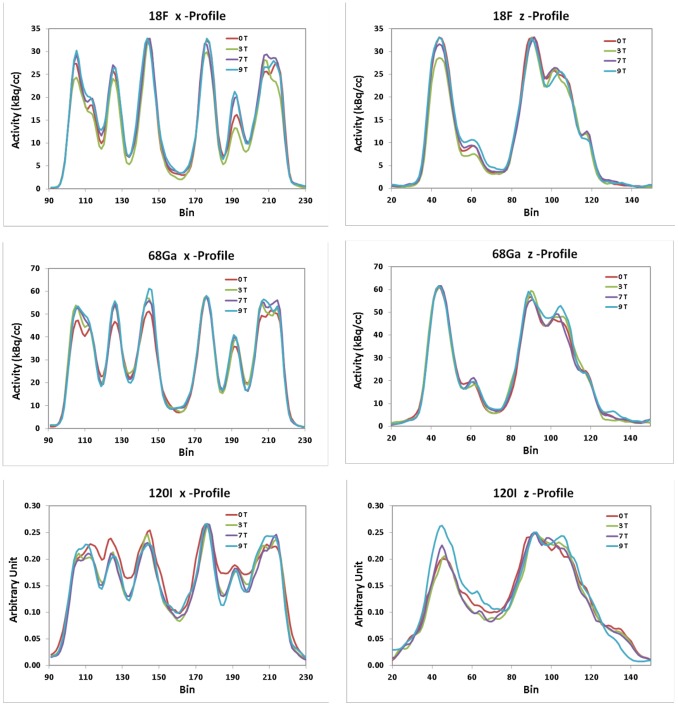
Profiles crossing the ‘basal ganglia’ in the x- and z-direction, i.e. perpendicular and along the magnetic field, respectively, were defined in images of the Iida brain phantom. The unit of the ^120^I data is arbitrary since these data are not scatter-corrected and not calibrated.

Since the magnetic field with its orientation along the z-axis reduces the positron range in the transversal (x-y) plane, images of the Iida brain phantom viewed in the coronal (x-z) or sagittal (z-y) plane are expected to show an improved resolution just in the x- or y-direction. This statement was experimentally confirmed by the results presented in Fig. 8 and 9.

Looking at coronal images of the Iida brain phantom filled with ^18^F, ^68^Ga or ^120^I there is no difference between the red contour, which represents the cortical outline at 0 T, and the green contour, which represents the cortical outline at 9.4 T (Fig. 8). Upon closer examination of the corresponding outlines in the case of the medium- and high-energy positron emitter, the red contour is outside the green contour in the horizontal direction of the image, which is oriented perpendicular to the magnetic field. For ^120^I this difference is greater. In the z-direction, the red and green outlines coincide for ^68^Ga and ^120^I. For ^120^I even a slight extension in z-direction becomes visible.

The peak-to-trough differences of the profiles through images of the Iida brain phantom are another indicator of changes in resolution and contrast when the phantom is measured at the different magnetic field strengths (Fig. 9). Profiles crossing the Iida brain phantom filled with ^18^F show no consistent differences between the different field strengths. This is in agreement with the practically unchanged GM/WM ratios for ^18^F (Table 3). In case of ^68^Ga, the x-profile at 0 T has a smaller peak-to-trough difference at 0 T compared to the other field strengths, whereas the z-profiles do not markedly differ. In the case of ^120^I, the x-profile shows a significantly smaller peak-to-trough difference at 0 T compared to 3 T, 7 T and 9.4 T; there was no consistent differences between the non-zero field strengths. For ^68^Ga and ^120^I the greatest peak-to-trough difference were found at 9.4 T.

## Discussion

This work presents experimental studies on the effects of the magnetic field, found in conventional high and ultra-high field MRI, on the positron range of different emitters. Using low-, medium- and high-energy positron emitters embedded in point and line sources as well as in a realistic brain phantom, a comprehensive overview of the MR-related influences on the positron range was obtained.

Visible inspection of the images obtained with the low-energy positron emitter, ^18^F, showed no magnetic field induced improvement in image resolution. This is in agreement with the numerical evaluation of the line source test, where no trend for an improved resolution from 0 T to 9.4 T was found in the case of ^18^F. The corresponding change of the GM/WM ratio measured in the Iida brain phantom was only 2%. These findings are in agreement with the very small change of FWHM from 3.85 mm at 0 T to 3.78 mm at 10 T reported by Raylman et al. [12] for detector crystals sized 5×5 mm^2^.

When line sources filled with ^68^Ga and ^120^I were measured, the image resolution improved with increasing magnetic field as seen in Fig. 6: this is supported by the numerical results in Table 2. Correspondingly, in the case of the Iida brain phantom, a better image resolution and contrast was observed already at 3 T, which improved further at 7 T and 9.4 T (Fig. 7). When comparing the findings of FWHM and FWTM in Table 2 with the GM/WM ratios reported in Table 3, one can conclude that the image improvement is more related to the smaller FWTM than to the smaller FWHM. Especially at 3 T and 7 T, the relative increases of GM/WM correspond better to the relative decreases of FWTM than of FWHM. As shown by Fig. 7, the images do not change significantly between 7 T and the 9.4 T. This agrees with the practically unchanged GM/WM ratio at 7 T and 9.4 T with values of 2.49±0.04 and 2.49±0.05 for ^68^Ga, and of 1.67±0.04 and 1.68±0.02 for ^120^I.

Images of the Iida brain phantom filled with ^68^Ga were slightly blurred compared to the ^18^F images when measured at 0 T, which improved at higher field strengths. Since the positron energy of ^68^Ga is similar to that of ^15^O, which is not so appropriate for the tests reported here due to its short half-life of just 2 min, our results indicate the improvement which can be expected in studies such as those described by the Tuebingen group which were performed with a small animal 7 T MR-PET system (personal communication). Especially in small animal MR-PET with an intrinsic PET-resolution of 1–2 mm, ^15^O studies will benefit from the influence of a strong magnetic field.

Images of the Iida brain phantom filled with ^120^I and measured at 0 T demonstrated a poor spatial resolution and contrast that would not be of much use in practical terms. Here, the advantage of the presence of a magnetic field during the PET acquisition became obvious. Even at 3 T the images demonstrated a marked improvement in resolution and contrast over those at 0 T. Further qualitative improvements were observed at 7 T and 9.4 T. These finding are supported by the ‘GM/WM’ ratios presented in Table 3. On the other hand, when examining the ^120^I images one must be aware that this positron emitter has a positron abundance of 56% and that ^120^I images have a flat background caused by the so-called gamma-coincidences [31]. If this background is corrected by an appropriate method such as described earlier [34], the quality of ^120^I images will improve outside and inside the MR environment – with the best overall image quality at 9.4T. With its half-life of 81 min and β^+^ abundance of 56%, ^120^I may be regarded as a short-lived substitute for the SPECT radioisotope ^123^I. In small animal PET data acquired simultaneously with high-field MR, it may be an interesting radiolabel for many radiotracers commonly applied in SPECT and radiolabelled with ^123^I. Even though the practical importance of ^120^I may be small, the results reported here can be regarded as representative for other high-energy positron emitter such as ^82^Rb (maximum positron energy 3.2 MeV) or ^76^Br (maximum positron energy 3.4 MeV).

The positron range of ^120^I has been considered only in one previous paper [35], whereas ^82^Rb is often examined in the literature as a typical high-energy positron emitter [12], [13], [15], [24], [36], [37]. ^82^Rb is typically used to examine myocardial flow. In this case the image resolution is not only affected by a long positron range, but also by the beating heart. Therefore, the expected increase of PET image resolution in a high-field MRI scanner should be complemented by an acquisition protocol that takes into account heart motion and rhythm by appropriate triggering.

The problem of motion in the case of a heart study alluded to above is a general cause of image blurring and becomes more relevant with increasing image resolution. In the context of the present study one must take account of patient motion by appropriate restriction or correction. Otherwise, the improvement of image resolution within the magnetic field remains ineffective.

The radiotracer distribution in the grey matter compartment of the Iida brain is similar to the uptake pattern observed in FDG or ^15^O-water studies. In spite of this, the images shown in our study do not mirror the complete situation in a clinical PET-MR study. In such a study there would be radiotracer uptake also in the white matter so that the GM/WM ratio would be dependent also on this uptake and not only on the reduced positron range like in our findings. Furthermore, in our study no head RF coils were present during the scan. In a real PET-MR study, the quality of the PET images may additionally be influenced by attenuation and scatter introduced by the head RF coils.

The Lorenz force induced by the magnetic field of the MRI scanner acts only in the plane perpendicular to the z-axis of the scanner. Therefore, one might naively expect no effect on the z-coordinate of images oriented along the z-axis. As reported in the literature [12], [15] and confirmed by our results shown in Fig. 5, point source images of the medium- and high- energy positron emitters exhibited tails in the z-direction when measured in the magnetic field. Positrons which leave the point source at angles oblique to the transaxial plane are deflected by the Lorenz force towards the z-axis so that their annihilation points are shifted towards the z-axis and become “visible” by the tails. The observation of the tails leads to the expectation that the MR field causes an elongation in z-direction together with a contraction in the transaxial plane in coronal or sagittal images of phantoms such as the Iida brain phantom. Figure 8 however, shows a slight elongation in z-direction in the ^120^I test only, but not for ^68^Ga. The z-profiles shown in Fig. 9 for ^68^Ga and ^120^I support this observation.

The restriction of the positron range in the plane perpendicular to the z-axis of the scanner is expected to turn a somewhat poorer isotropic image resolution, which is at least found at the centre of the field-of-view, into an anisotropic one. Consequently, the partial volume effect in the x-y plane should be less than in the x-z and y-z planes. This might influence image analysis, for example, if the patient’s head is differently tilted in two consecutive scans so that planes affected by the restriction of the positron range do not have the same orientation. However, when looking at Figure 7, which presents the images of the irregularly shaped brain phantom, it is very difficult to realise that the restricted positron range leads to an improvement in resolution and contrast of the image of cortical structures only in x- and y-direction. PET images do not comprise an ideal plane, but always a slice containing three-dimensional voxels. The value of an image voxel is influenced by all three dimensions; two of them are affected by the magnetic field and one not. The same pertains to volumes of interest with many voxels, which are commonly applied in data analysis. Thus, effects of image resolution and related partial effects differing for x, y, and z are mixed when averaging the voxels of a VOI.

## Conclusions

The use of an integrated, hybrid MR-PET scanner enables the exploitation of advantages accruing from the investigation of different processes and functions observable either by MRI or by PET simultaneously such that complementary results can be obtained. Additionally, the MR field helps to improve the resolution and contrast of PET images if the applied radiotracer is labelled with a medium- or high-energy positron emitter. Although the best improvement was found with an ultra-high field MRI system, our data show an improvement already at 3 T, the field strength of the first commercial integrated MR-PET scanner for human studies. Whilst no commercial integrated human MR-PET scanner with a high-field MR magnet (>3 T) is available, an integrated small animal MR-PET operating at a field of 7 T has been developed and commercial devices are expected in the near future. PET imaging in such systems where resolution might be even more crucial would benefit from the co-existing MR field.
